# Crosstalk between adipose-derived stem cells and chondrocytes: when growth factors matter

**DOI:** 10.1038/boneres.2015.36

**Published:** 2016-02-03

**Authors:** Juan Zhong, Bin Guo, Jing Xie, Shuwen Deng, Na Fu, Shiyu Lin, Guo Li, Yunfeng Lin, Xiaoxiao Cai

**Affiliations:** 1 State Key Laboratory of Oral Diseases, West China Hospital of Stomatology, Sichuan University, Chengdu 610041, China; 2 Institute of Stomatology, General Hospital of Chinese PLA, Beijing 100853, China

## Abstract

Adipose-derived stem cells (ASCs) and mesenchymal stem cells are promising for tissue repair because of their multilineage differentiation capacity. Our previous data confirmed that the implantation of mixed ASCs and chondrocytes into cartilage defects induced desirable *in vivo* healing outcomes. However, the paracrine action of ASCs on chondrocytes needs to be further elucidated. In this study, we established a co-culture system to achieve *cell-to-cell* and *cell-to-tissue* crosstalk and explored the soluble growth factors in both ASCs and chondrocytes supplemented with 1% fetal bovine serum to mimic the physiological microenvironment. In ASCs, we screened for growth factors by semi-quantitative PCR and quantitative real-time PCR and found that the expression of bone morphogenetic protein 2 (BMP-2), vascular endothelial growth factor B (VEGFB), hypoxia inducible factor-1α (HIF-1α), fibroblast growth factor-2 (FGF-2), and transforming growth factor-β1 significantly increased after co-culture in comparison with mono-culture. In chondrocytes, VEGFA was significantly enhanced after co-culture. Unexpectedly, the expression of collagen II and aggrecan was significantly down-regulated in the co-culture group compared with the mono-culture group. Meanwhile, among all the growth factors screened, we found that the BMP family members BMP-2, BMP-4, and BMP-5 were down-regulated and that VEGFB, HIF-1α, FGF-2, and PDGF were significantly decreased after co-culture. These results suggest that crosstalk between ASCs and chondrocytes is a pathway through the regulated growth factors that might have potential in cartilage repair and regeneration and could be useful for tissue engineering.

## Introduction

Cartilage is an avascular tissue and not able to heal spontaneously; thus, the nutrient supply for chondrocytes depends predominantly on synovial diffusion.^1^ Tissue engineering holds promise for the healing of damaged tissues and organs by manipulating cells, scaffolds, and stimuli in order to solve the problems resulting from severe trauma, resection of tumors, and congenital deformity.^2^ Many approaches have been developed for cartilage regeneration, including the implantation of cells and tissues such as autologous chondrocytes^3,4^ with or without biomaterials.^5,6^ Although chondrocytes are a promising candidate for chondrogenesis and widely applied in cartilage regeneration, a limited cell number can be obtained from autologous cartilage. Therefore, other cell types, such as stem cells, have been identified as interesting alternative sources.^7^ Adipose-derived stem cells (ASCs), a promising multipotential candidate for tissue engineering, have been widely utilized for chondrogenesis,^8^ osteogenesis,^9^ and adipogenesis.^10^

Methods for co-culturing multiple cell types, such as pellet three-dimensional culture,^11,12^ and non-contact co-culture,^13^ are used to study the influence of the cell complex on tissue regeneration. Pellets of mesenchymal stem cells (MSCs) and chondrocytes can stimulate cartilage formation, independently of culture conditions and cell sources,^14^ and enhance matrix formation and expression of collagen I mRNA^15^ because of their tropic effects on chondrocytes. In our previous study, ASCs and chondrocytes were implanted into knee defects, resulting in cartilage healing.^16^ However, little is known about the mechanism for the enhancement of chondrogenesis and the growth factors secreted by ASCs and chondrocytes.

Our current study focuses on growth factors to better understand the influence of co-culture on each cell type and to provide evidence for the co-culture assay. By confirming the key growth factors involved in promoting chondrogenesis, we hope to gain insight to the paracrine signaling component of cartilage repair and regeneration.

## Materials and methods

### Isolation and culture of rat ASCs and chondrocytes

The animal materials used for this study were obtained according to ethical principles, and the protocol was reviewed and approved by our Institutional Review Board.

Adipose cells were taken from the subcutaneous adipose tissue of 5-day-old female rats. ASCs were isolated and cultured as previously described.^17^ Briefly, the fat was cut into small pieces and digested with collagenase I at 37 °C for 45 min. Enzyme activity was neutralized with α-glucose Dulbecco’s Modified Eagle’s Media (α-MEM; Hyclone, Pittsburgh, USA) supplemented with 10% fetal bovine serum (FBS) and 100 U·mL^−1^ penicillin/streptomycin (Hyclone). The mixed suspension was centrifuged at 200*g* for 5 min, and then the supernatant was removed. Ten percent of FBS α-MEM was added to resuspend the ASCs. Then, the ASCs were seeded in T25 culture flasks and cultured at 37 °C, under a 5% CO_2_ atmosphere, until passage II, when purified ASCs could be obtained.

The chondrocytes and cartilage explants were isolated from the knee cartilage of approximately 3-day-old rats. For chondrocyte culturing, briefly, cartilage was minced into small pieces and pretreated with 0.25% trypsin for 30 min, and then washed three times with phosphate-buffered saline (PBS) to eliminate the trypsin. Cartilage pieces were digested with collagenase type II (0.1%) for 2–3 h in a 37 °C water bath. After centrifugation at 200*g* for 5 min, the mixed cartilage and chondrocytes were suspended in DMEM supplemented with 10% FBS and 100 U·mL^−1^ penicillin/streptomycin. Then, the chondrocytes were seeded in T25 culture flasks and cultured at 37 °C, under a 5% CO_2_ atmosphere, until passage II for usage.

### Co-culture of ASCs and chondrocytes or cartilage explants

The gene profiles of soluble growth factors in ASCs and chondrocytes were detected by semi-quantitative PCR under trans-well co-culture. ASCs or chondrocytes were seeded at 1 × 10^4^ cells per cm^2^ (six-well plates, Corning, New York, USA) on 0.4 μm porous membranes or the lower chamber. ASCs and chondrocytes were cultured in DMEM and α-MEM media, respectively. Once the cells were adherent to plates and equilibrated for 24 h, the culture media were replaced with 2% FBS media for a 12 h starvation. After starvation, the media were changed to fresh 1% FBS DMEM.

### Cell proliferation and migration detection

To detect the cell proliferation and migration index in the mono- and co-culture groups, passage II ASCs and chondrocytes were seeded onto the upper or lower chambers in E-plates at 1–5 × 10^3^ cells per well. After 24 h, data were recorded by real-time cell analyzer (RTCA) iCELLigence (ACEA Biosciences, San Diego, CA, USA) connected to a computer.

### Immuno-fluorescent staining

To immunostain the cells passed through the wells, the cells were seeded onto the upper chambers with an 8 μm porous membrane in six-well plates. After 24 h, the cells on the underside of the bottom were fixed with 4% paraformaldehyde for 10 min, and then the nuclei were stained with 4′,6-diamidino-2-phenylindole (DAPI) for another 10 min. Images were taken with an inverted fluorescence microscope (Olympus, Tokyo, Japan).

To immunostain the cytoskeleton, the cells were fixed with 4% paraformaldehyde for 20 min and washed with PBS two times. The cells were blocked with 5% bovine serum albumin for 60 min before being incubated with fluorescein isothiocyanate -phalloidin at a 1:100 dilution. Then, the samples were stained with DAPI for 10 min, and images were captured by microscope.

### Semi-quantitative PCR

RNA was collected from cultured ASCs and chondrocytes after co-culturing for 1, 2, 3, 5, and 7 d using the RNeasy Plus Mini Kit (Qiagen, Venlo, Netherlands) with a genomic DNA eliminator. The purified RNA was dissolved in RNase-free water, then 2 μL of the RNA solutions were used to measure the optical density at 260 nm with a spectrophotometer. The RNA samples were treated with DNase I (Mbi, Maryland, MD, USA), and cDNA was prepared from each sample using the cDNA synthesis kit (Mbi) in a final volume of 20 µL. Semi-quantitative PCR was performed with a PCR kit (Mbi) in a 25 µL volume containing a 1 µL cDNA sample using a thermo-cycler (Bio-Rad, Hercules, CA, USA). The primers (Table 1) were searched by Basic Local Alignment Search Tool, and glyceraldehyde-3-phosphate dehydrogenase (*GAPDH*) and beta-actin (*β-Actin*) were used as the internal control and normalized standard, respectively. In the PCR program, cDNA was denatured for 5 min at 94 °C, followed by 30 cycles of 15 s at 94 °C, 15 s at 55–60 °C, and 30 s at 72 °C. The DNA was resolved into bands by electrophoresis on a 2% agarose gel in Tris/Borate/EDTA buffer, which represented the expressing dose of the soluble growth factor genes, and the bands were visualized by staining with GoldView (Heart, Shanghai, China).

### Quantitative real-time PCR

Quantitative real-time PCR was performed with the SYBR *Premix Ex Taq* II PCR Kit (TAKARA, Shiga, Japan) using an iCycler (Bio-Rad) according to the manufacturer’s protocol. The PCRs contained 1.0 μmol⋅L^−1^ of the forward or reverse primers (Table 1) and 2 μL sample cDNA in a 25 μL final volume. The reaction was initiated by activating the polymerase with a 5 s pre-incubation at 95 °C. Amplification was achieved with 39 cycles of 5 s denaturation at 95 °C, 30 s annealing at 60 °C, and 5 s extension at 72 °C. All experiments were performed in triplicate. The copy numbers of each gene were determined by cycle threshold (△CT) methods. The means of the copy numbers of GAPDH were used as internal controls to normalize the data.

### Statistical analysis

All experiments were performed in triplicate and reproduced at least three separate times. Statistical analysis of the data was performed with SPSS 16.0 using independent sample *t*-test analysis to determine whether differences existed. The critical significance level was set to *P* < 0.05.

## Results

### Morphological features and cell behavior characteristics of ASCs and chondrocytes were influenced by the co-culturing conditions

**Figure 1:**
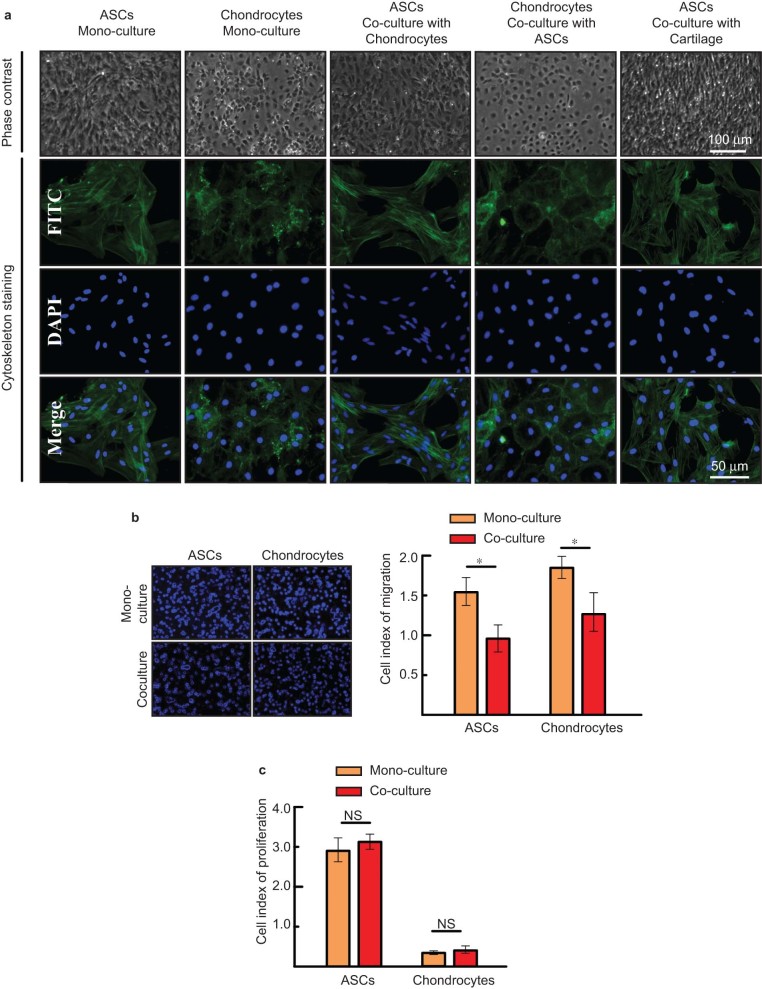
Cell morphologies and cell behaviors of ASCs and chondrocytes. (**a**) Cell morphologies and cytoskeleton staining of ASCs and chondrocytes after 7 days of mono- and co-culture. Cell morphological features are shown in phase-contrast (upper) and with immune-fluorescent staining for the cytoskeleton (lower). (**b**) Cell migration assay of ASCs and chondrocytes by trans-well chamber (left lane). Cell migration rates after co-culture at 24 h by RTCA confirmed the results by trans-well chamber. *P < 0.05, compared with the mono-culture control. The data were represented as the means ± SD (*n* = 3). (**c**) Cell proliferation of ASCs and chondrocytes after co-culture, detected by RTCA. No significant differences were found between the mono- and co-culture groups. NS denotes no significant difference. The data were represented as the means ± SD (*n* = 3).

The cell morphologies, observed with phase-contrast microscopy, and the cytoskeleton, visualized with immune-fluorescent staining, indicated that the cells cultured with low-density serum in the media maintained normal cellular vitality and complete cellular integrity (Figure 1a). The migration and proliferation curves were obtained for the ASCs and chondrocytes after 36 h in mono- or co-culture (data not shown). At 24 h, the amount of ASCs and chondrocytes in the upper 8.0 μm well passing through the porous membrane was determined by DAPI nuclear staining (Figure 1b). The cell index of migration showed that the migration of ASCs or chondrocytes in the co-culture experiment was significantly decreased after 24 h, which may demonstrate crosstalk between ASCs and chondrocytes (Figure 1b). However, cell proliferation was not significantly different between the co-culture and mono-culture groups (Figure 1c).

### Co-culture with ASCs down-regulates specific genes in chondrocytes

**Figure 2:**
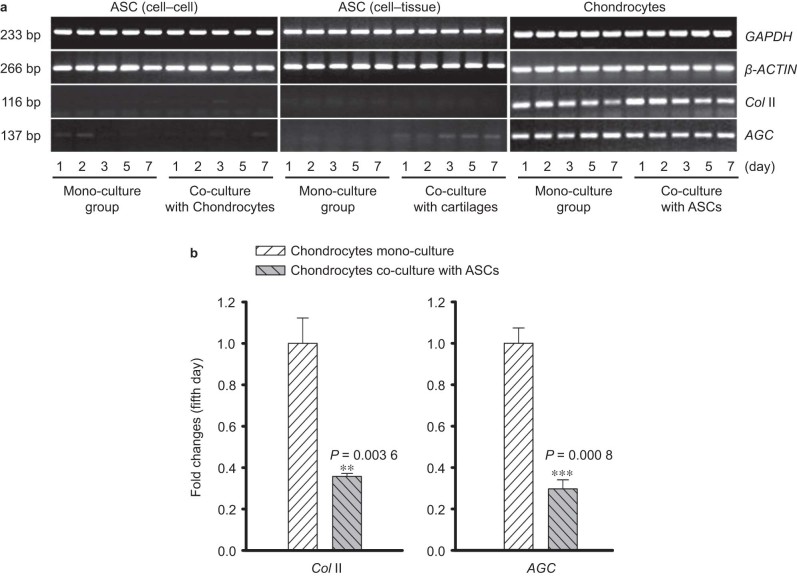
Co-culture with ASCs down-regulates specific genes in chondrocytes. (**a**) *Col* II and *AGC* in ASCs and chondrocytes after mono- and co-culture, as detected by semi-quantitative PCR. Glyceraldehyde-3-phosphate dehydrogenase (*GAPDH*) and *β-Actin* were used as the internal controls. The gels shown are representative of three different experiments (*n* = 3). The cultured cell samples were taken at 1, 2, 3, 5, and 7 d. (**b**) Quantitative real-time PCR confirmed the different expression of *Col* II and *AGC* in both ASCs and chondrocytes at the fifth day. *GAPDH* was used as the internal control. The △Ct method was used to measure the fold changes. The data presented are the means of three different experiments (*n* = 3). *Col* II and *AGC* show no expression in ASCs but lower expression in chondrocytes co-cultured with ASCs. ***Represents *P* < 0.001. The error bar reflects the SD.

The specific gene expression of the chondrogenic markers, *Col* II and *AGC*, were investigated by semi-quantitative PCR (Figure 2a) and quantitative real-time PCR (Figure 2b) to determine how the differentiation of ASCs and chondrocytes is influenced by co-culture. In ASCs, no expression of *Col* II and *AGC* was detected at the fifth day. Compared with chondrocytes in mono-culture, *Col* II and *AGC* expression after co-culture with ASCs were down-regulated to 35.8% and 29.7%, respectively (Figure 2b). These findings might indicate a dedifferentiated performance of chondrocytes influenced by co-culture with ASCs.

### BMP family is modulated in both ASCs and chondrocytes by co-culture

**Figure 3:**
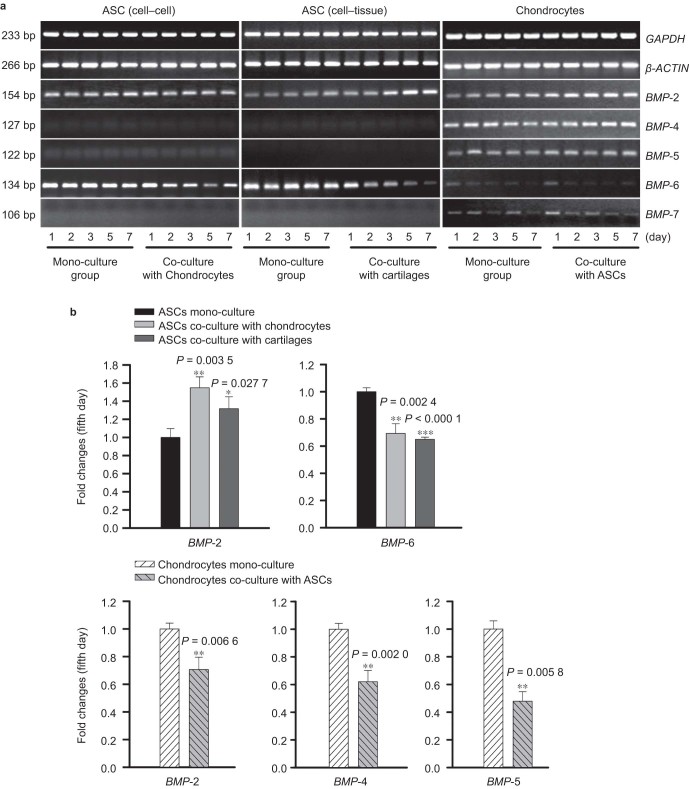
The BMP family is modulated in both ASCs and chondrocytes after co-culture. (**a**) Variation in gene expression of the BMP family members, *BMP*-2, *BMP*-4, *BMP*-5, *BMP*-6, and *BMP*-7, in ASCs and chondrocytes after mono- and co-culture, is revealed by semi-quantitative PCR. *GAPDH* and *β-Actin* were used as the internal controls. The gels shown are represent ative of three different experiments (*n* = 3). The cultured cell samples were taken from 1, 2, 3, 5, and 7 d. (**b**) Quantitative real-time PCR confirmed the different expression of *BMPs* in both ASCs and chondrocytes. GAPDH was used as the internal control. The △Ct method was used to calculate the fold changes. The data presented are the means of three different experiments (*n* = 3). *BMP*-4/-5/-7 in ASCs and *BMP*-6/-7 in chondrocytes showed no expression, consistent with the results of semi-quantitative PCR. The cultured cell samples were taken on the fifth day. The *, **, and *** represent *P* < 0.05, *P* < 0.01, and *P* < 0.000 1, respectively. The error bar reflects the SD.

Gene expression of the BMP family members *BMP*-2, *BMP*-4, *BMP*-5, *BMP*-6, and *BMP*-7 was examined by semi-quantitative PCR (Figure 3a) and quantitative real-time PCR (Figure 3b). The expression of *BMP*-2 in ASCs co-cultured with chondrocytes and cartilage explants was increased 1.549-fold and 1.318-fold, respectively, while *BMP*-6 expression was down-regulated to 69.3% and 65.0%, respectively. In chondrocytes, *BMP*-2, *BMP*-4, and *BMP*-5 were down-regulated in the co-culture group (to 70.7%, 62.1%, and 47.9%, respectively) (Figure 3b).

### VEGF members and vascular-related growth factors were regulated in both ASCs and chondrocytes after co-culture

**Figure 4:**
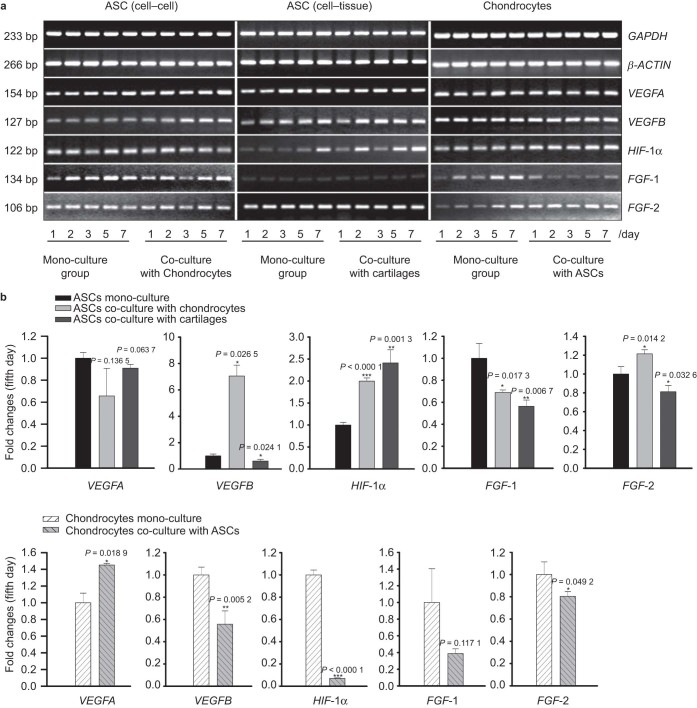
VEGF members and vascular-related growth factors are regulated in both ASCs and chondrocytes after co-culture. (**a**) The *VEGFs* include *VEGFA* and *VEGFB* and the *FGFs* include *FGF-1* and *FGF-2*. *VEGFs*, *HIF*-1α, and *FGFs* in ASCs and chondrocytes after mono- and co-culture were detected by semi-quantitative PCR. GAPDH and β-Actin were used as the internal controls. The gels shown are representative of three different experiments (*n* = 3). The cultured cell samples were taken from 1, 2, 3, 5, and 7 d. (**b**) Quantitative real-time PCR confirmed the differential expression of *VEGFs*, *HIF*-1α, and *FGFs* in both ASCs and chondrocytes on the fifth day. *GAPDH* was used as the internal control. The △Ct method was used to calculate the fold changes. The data presented are the means of three different experiments (*n* = 3). The *, **, and *** represent *P* < 0.05, *P* < 0.01, and *P* < 0.000 1, respectively. The error bar reflects the SD.

VEGFA/B and the vascular-related growth factors, *HIF*-1α, fibroblast growth factor**-**1 (*FGF*-1), and *FGF*-2, were detected by semi-quantitative PCR (Figure 4a) and quantitative real-time PCR (Figure 4b). VEGFB expression significantly increased in ASCs co-cultured with chondrocytes, with changes up to 5.757-fold; meanwhile the expression decreased in ASCs co-cultured with cartilage explants (down to 60.9%) compared with that in ASCs in mono-culture. Moreover, we found that *HIF*-1α expression was significantly up-regulated in ASCs co-cultured with chondrocytes (up to 1.998-fold) and cartilage explants (up to 2.412-fold) compared with mono-cultures. After co-culturing with the chondrocytes and cartilage explants, *FGF*-1 expression in ASCs decreased (down to 68.9 and 56.4%, respectively). In addition, *FGF*-2 expression increased 1.214-fold in ASCs co-cultured with chondrocytes, but decreased to 81.2% in ASCs co-cultured with cartilage explants. In chondrocytes, *VEGFA* expression was up-regulated to 1.452-fold after co-culture with ASCs, while *VEGFB*, *HIF*-1α, and *FGF*-2 expression decreased to 55.8, 7.1, and 80.4%, respectively. However, *FGF*-1 showed no significant changes in chondrocytes after co-culture (Figure 4b).

### Relevant growth factors varied expression in ASCs and chondrocytes after co-culture

**Figure 5:**
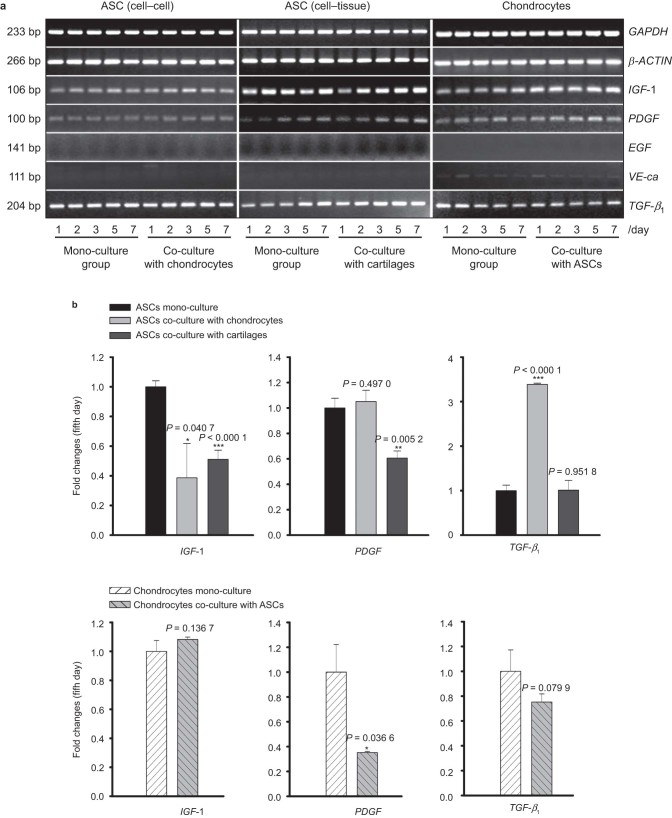
Relevant growth factors have varied expression in ASCs and chondrocytes after co-culture. (**a**) Semi-quantitative PCR revealed variations in *IGF*-1, *PDGF*, *EGF*, *VE-ca*, and *TGF-β_1_* in ASCs and chondrocytes after mono- and co-culture. *GAPDH* and *β-Actin* were used as the internal controls. The gels shown are representative of three different experiments (*n* = 3). The cultured cell samples were taken from 1, 2, 3, 5, and 7 d. (**b**) Quantitative real-time PCR confirmed the differential expressions of relevant growth factors in both ASCs and chondrocytes on the fifth day. The △Ct method was used to calculate the fold changes. The data presented are the means of three different experiments (*n* = 3). *EGF* and *VE-ca* showed no expression in ASCs and chondrocytes. The *, **, and *** represent *P* < 0.05, *P* < 0.01, and *P* < 0.000 1, respectively. The error bar reflects the SD.

We then investigated the gene expression of insulin-like growth factor (*IGF*), *PDGF*, *VE-ca*, *EGF*, and *TGF*-β_1_, which are related to chondrogenesis, in ASCs and chondrocytes by semi-quantitative PCR (Figure 5a) and quantitative real-time PCR (Figure 5b). *VE-ca* and *EGF* were not expressed in ASCs and chondrocytes. *TGF*-*β*_1_ expression increased 3.387-fold while *IGF*-1 expression in ASCs co-cultured with chondrocytes decreased to 38.7% compared with that in ASC mono-cultures. Additionally, PDGF expression in ASCs showed no significant variation between groups. Co-culture with cartilage explants resulted in significantly lower *IGF*-1, *PDGF*, and *TGF*-*β*_1_ expression compared with ASC controls (55.1, 60.6, and 50.5% lower, respectively). In chondrocytes co-cultured with ASCs, *PDGF* expression was down-regulated to 35.2% compared with chondrocytes in mono-culture. However, *IGF*-1 and *TGF*-*β*_1_ expressions were not significantly different (Figure 5b).

## Discussion

The interactions between transplanted cells and local chondrocytes or cartilage explants in cartilage defects have a critical role in the efficacy of cell-based therapy for cartilage repair. The paracrine influence of ASCs on chondrocytes is unclear, as is how such interactions influence the resulting cartilage repair. As cartilage is an avascular tissue, the effects on ASCs and chondrocytes in co-culture circumstances supplemented with 1% FBS need further investigation. Therefore, by screening a large gene expression profile for soluble growth factors related to chondrogenesis and cell differentiation in different culture conditions, we established a novel insight into the crosstalk between ASCs and chondrocytes.

The overall study showed that the soluble growth factors in ASCs and chondrocytes significantly varied after co-culture, compared with mono-culture. In ASCs, no expression of *Col* II and *AGC* was detected at the fifth day, implying that the early chondrogenic commitment of ASCs needs further investigation. To mimic physiological conditions, we established a low serum concentration of 1% and found that *Col* II and *AGC* expression in chondrocytes after co-culture with ASCs was down-regulated, in contrast to the results of an obvious increasing up-regulation in the performance of cartilage matrix formation.^11,18^ In addition, cell morphologies were also visualized, revealing notable changes after co-culture. Growth factors from endogenic production are essential for cell migration and proliferation.

ASCs are promising for tissue regeneration because they can be differentiated into chondrocytes,^19^ osteoblasts,^20,21^ endothelial cells^22^ and so on. Co-cultured pellets of MSCs and chondrocytes can stimulate cartilage formation.^14^ Our previous study found that cells seeded onto the scaffolds showed better adhesion, migration, and proliferation and that co-cultured cell-based *TGF*-*β*_1_/scaffolds revealed desirable healing outcomes *in vivo*.^16^ *BMP*-2 and *TGF*-*β*_1_, as regulators of chondrogenesis and chondrogenic hypertrophy,^23,24^ may have an important role in the deposition of cartilage extracellular matrix through the crosstalk between *TGF* and *BMP* signaling.^25^ In our study, *TGF*-*β*_1_ in ASCs co-cultured with chondrocytes was up-regulated, consistent with previous studies. Moreover, *BMP*-2 expression was higher in ASCs co-cultured with chondrocytes or cartilage explants. The secreted soluble factors, *VEGF*,^26^ *HIF*-1α, *IGF*-1,^27,28^ and fibroblast growth factor (*FGF*),^29^ that were confirmed in ASCs and chondrocytes have a close relationship to early chondrogenesis and chondrogenic differentiation. *HIF*-1α deficiency may affect chondrogenesis of ASCs *in vitro*.^30^ *FGF*-2 primes cells for chondrogenesis during *in vitro* expansion.^31^ We observed that *HIF*-1α and *FGF*-2 expression both dramatically increased after co-culture.

Some limitations apply to this study. Many growth factors that we did not consider could also be involved in crosstalk between ASCs and chondrocytes. Moreover, the effects of each growth factor on ASCs and chondrocytes are still unknown and should be further confirmed. Therefore, microarray analysis may be applied to detect changes in the gene profile to confirm these results in the future. We intend to identify the factors that can strengthen chondrogenesis and may be applied to clinical treatments.

## Conclusion

Taken together, our data indicate that the soluble growth factors secreted by ASCs and chondrocytes varied significantly after co-culture. Cell proliferation and migration and cell morphologies were influenced by the non-contact communication. Crosstalk between ASCs and chondrocytes is a path through the regulated growth factors that could be useful in cartilage repair and regeneration and could be exploited for tissue engineering. Further studies will be required to detect more key factors involved in the co-culture system.

## Figures and Tables

**Table 1:** Forward and reverse sequences of primers designed for the analysis of internal controls and related growth factor genes by quantitative real-time PCR

mRNA	Primer pairs
*GAPDH* (233 bp)	Forward ACAGCAACAGGGTGGTGGAC
	Reverse TTTGAGGGTGCAGCGAACTT
*β-ACTIN* (266 bp)	Forward CACCCGCGAGTACAACCTTC
	Reverse CCCATACCCACCATCACACC
*Col* II (116 bp)	Forward TCAAGTCGCTGAACAACCAG
	Reverse GTCTCCGCTCTTCCACTCTG
*AGC* (137 bp)	Forward GCAGCACAGACACTTCAGGA
	Reverse CCCACTTTCTACAGGCAAGC
*BMP*-2 (102 bp)	Forward TCAAGCCAAACACAAACAGC
	Reverse CCACGATCCAGTCATTCCA
*BMP*-4 (101 bp)	Forward GACTTCGAGGCGACACTTCT
	Reverse AGCCGGTAAAGATCCCTCAT
*BMP*-5 (115 bp)	Forward AAGGAGGCTTGGGAGACAAT
	Reverse CTGTGAGGCAAACCCAGAAT
*BMP*-6 (101 bp)	Forward TGTCAGAGGGAGAGGGACTG
	Reverse CTTGCGGTTCAGGGAGTGT
*BMP*-7 (197 bp)	Forward CGCTCCAAGACTCCAAAGAA
	Reverse TTCAGAGGGAAGGCACACTC
*VEGFA* (154 bp)	Forward TCATCAGCCAGGGAGTCTGT
	Reverse TGAGGGAGTGAAGGAGCAAC
*VEGFB* (127 bp)	Forward GCAACACCAAGTCCGAATG
	Reverse TGGCTTCACAGCACTCTCC
*HIF*-1α (122 bp)	Forward CGATGACACGGAAACTGAAG
	Reverse CAGATTCAGGTAATGGAGACA
*FGF*-1 (134 bp)	Forward GGCTCGCAGACACCAAAT
	Reverse CGCTTACAACTCCCGTTCTT
*FGF*-2 (106 bp)	Forward CCATCAAGGGAGTGTGTGC
	Reverse TCCAGGCGTTCAAAGAAGAA
*IGF*-1 (106 bp)	Forward TCTACCTGGCACTCTGCTTG
	Reverse GGTCCACACACGAACTGAAG
*PDGF* (100 bp)	Forward GCTGTTCACTTGCTTCTTGC
	Reverse AGGCACCACTTCCATTTCTG
*EGF* (141 bp)	Forward GCCACGGTTACATTCACTCC
	Reverse TCCAAATCGCCTTCTCTTTC
*VE-ca* (111 bp)	Forward ACGAGGACAGCAACTTCACC
	Reverse GCACAGGCAGGTAGTGGAAC
*TGF-β*_1_ (204 bp)	Forward CCGCAACAACGCAATCTAT
	Reverse CCAAGGTAACGCCAGGAAT
